# The Impact of the Coronary Artery Calcium Score on the Clinical Outcomes in Patients with Acute Myocardial Infarction

**DOI:** 10.3390/jcm13237136

**Published:** 2024-11-25

**Authors:** Hisashi Sato, Kenichi Sakakura, Hiroyuki Jinnouchi, Yousuke Taniguchi, Kei Yamamoto, Takunori Tsukui, Masashi Hatori, Taku Kasahara, Yusuke Watanabe, Shun Ishibashi, Masaru Seguchi, Hideo Fujita

**Affiliations:** Division of Cardiovascular Medicine, Saitama Medical Center, Jichi Medical University, 1-847 Amanuma, Omiya, Saitama City 330-8503, Japan

**Keywords:** coronary artery calcium score, acute myocardial infarction, major adverse cardiovascular events

## Abstract

**Background**: It is essential to identify the risk factors for poor clinical outcomes in patients with acute myocardial infarction (AMI). The coronary artery calcium score (CACS) is gathering attention as a predictor for future cardiovascular events. This study aimed to (1) measure CACSs in patients with AMI by non-ECG-gated computed tomography (CT), (2) compare clinical outcomes between patients with a high CACS and a low–intermediate CACS and (3) to elucidate the association between high CACS and clinical outcomes. **Methods**: We defined the high CACS group as the highest quantile of CACS (Q4) and defined the low–intermediate CACS group as the other quantiles of CACS (Q1–Q3). The primary endpoint was major adverse cardiovascular events (MACE), which were defined as the composite of all-cause death, re-admission for heart failure, non-fatal MI and target vessel revascularization. We included 548 patients with AMI who underwent non-ECG-gated CT and divided them into the high CACS group (CACS ≥ 5346.5, *n* = 137) and the low–intermediate CACS group (CACS ≤ 5329.3, *n* = 411). **Results**: During the median follow-up duration of 535 days, 150 MACE were observed. The Kaplan–Meier curves showed that MACE occurred more frequently in the high CACS group than in the low–intermediate CACS group (*p* < 0.001). Multivariable Cox hazard analysis revealed that a high CACS was significantly associated with MACE (hazard ratio 1.597, 95% confidence interval 1.081–2.358, *p* = 0.019) after controlling for multiple confounding factors. **Conclusions**: Clinical outcomes were worse in AMI patients with a high CACS than in those with a low–intermediate CACS. A high CACS was significantly associated with MACE in multivariate analysis.

## 1. Introduction

In developed countries, the mortality rate is high in patients with coronary artery disease, particularly with acute myocardial infarction (AMI) [1,2,3]. Some patients still have poor clinical outcomes, although the development of primary percutaneous coronary intervention (PCI) and optimal medical therapy has improved overall clinical outcomes of AMI [4,5]. However, the risk factors for mid-term or long-term clinical outcomes have not been established in patients with AMI. It is important to find patients with the risk factors for mid-term or long-term outcomes, which may lead to the efficient usage of limited medical resources [6].

In recent years, the coronary artery calcium score (CACS) has been a focus as a predictor for future cardiovascular events [7,8,9,10]. The CACS may be a predictor for clinical events in patients with AMI. However, the CACS has not been fully discussed in patients with AMI, partly because patients with AMI did not undergo chest plain computed tomography (CT) routinely until the pandemic of Coronavirus disease 2019 (COVID-19). During the pandemic of COVID-19, patients with AMI often underwent chest plain CT in emergency departments as a screening tool for pneumonia [11,12]. Consequently, it became possible to calculate CACS in those patients with AMI. This retrospective study aimed to compare clinical outcomes between patients with a high CACS and with a low–intermediate CACS and to find the association between a high CACS and clinical outcomes.

## 2. Methods

### 2.1. Coronary Artery Calcium Score

Since the COVID-19 pandemic, plain chest CT has been an important screening tool for pneumonia, especially in the emergency room. Plain chest CT also provides secondary information regarding pericardial effusion, aortic dissection and aortic aneurysm. Therefore, we routinely performed plain chest CT at admission or before primary PCI so as not to miss pneumonia, pericardial effusion, aortic dissection and aortic aneurysm. The CACS was obtained from non-electrocardiographic (ECG)-gated plain CT with a thickness of 0.5 mm, using Aquilion One (80-row or 320-row), Aquilion Prime (80-row), or Aquilion Prime SP (80-row) (Canon, Tokyo, Japan). The tube voltage was 120 kV. The image reconstruction method was the Precise IQ Engine, which was developed by Canon Medical. The scans were analyzed on SYNAPSE VINCENT Workspace (Fujifilm, Tokyo, Japan). The CACS was calculated as described by Agatston et al. [7].

### 2.2. Study Patient

We screened all patients with AMI at our institution between May 2020 and June 2023. The inclusion criterion was (1) patients with AMI. The exclusion criteria were (1) patients who have a history of coronary artery bypass graft surgery, (2) patients who received coronary artery stent, (3) patients who received pacemaker implantation, (4) patients who did not undergo plain chest CT, (5) patients who did not undergo PCI to the culprit lesion of AMI, (6) patients whose CACS was not available due to artifacts and (7) a second or more than second AMI during the study period, i.e., when a patient experienced ≥ 2 AMI during the study period.

The primary endpoint was major adverse cardiovascular events (MACE), which were defined as the composite of all-cause death, re-admission for heart failure, non-fatal MI and target vessel revascularization. We defined the high CACS group as the highest quantile of CACS (Q4) and defined the low–intermediate CACS group as the other quantiles of CACS (Q1–Q3). The study patients were divided into the high CACS group and the low–intermediate CACS group according to the above definition. According to this definition, patients who had a score of ≥5346.5 were assigned to the high CACS group, and patients who had a score of ≤5329.3 were assigned to the low–intermediate CACS group. These cut-off values are quite different from those used in previous studies. The previous studies adopted ECG-gated CT with a thickness of 3.0 mm slices, whereas we adopted non-ECG-gated CT with a thickness of 0.5 mm. The day of admission to our hospital was defined as the index day (day 1). The patients were followed until the point of MACE or until the study end date (4 June 2024).

### 2.3. Definitions

The universal definition was used for the diagnosis of AMI [13,14]. The definitions of background diseases are described elsewhere [15,16]. We used the laboratory data at admission [17]. Left ventricular ejection fraction (LVEF) was measured using transthoracic echocardiography during the index hospitalization. We also calculated the estimated glomerular filtration rate (eGFR) [18]. The initial and final TIMI flow grades were recorded based on the coronary angiography results [19].

### 2.4. Statistical Analysis

Percentages for categorical variables and the median (quartile 1–quartile 3) for nonparametric variables were used. The chi-square test was used for categorical variables. Continuous variables were compared using a Mann–Whitney U test. The Kaplan−Meier curves were drawn, and the log–rank test was used to find statistical differences between curves. We also performed a multivariate Cox hazard analysis to investigate the association between a high CACS and MACE after controlling for confounding factors. Variables that were significantly different (*p* < 0.05) between the high CACS and low–intermediate CACS groups were included as confounding factors. Variables with missing values were not included in the model. Furthermore, when there were ≥2 similar variables, only one variable was entered into the model to avoid multicollinearity. Multicollinearity was evaluated by the variance inflation factor (VIF). The hazard ratio (HR) and 95% confidence interval (CI) were calculated. A *p*-value < 0.05 was considered statistically significant. All analyses were performed using statistical software, SPSS version 24.0/Windows (SPSS, Chicago, IL, USA).

## 3. Results

**Figure 1:**
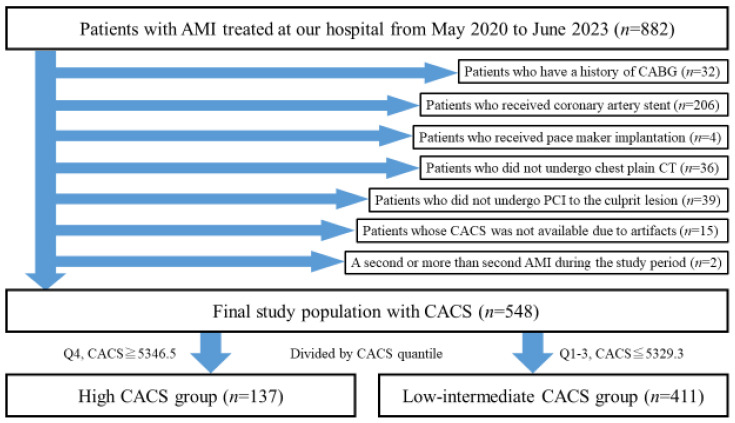
The study flow chart. Abbreviations: AMI = acute myocardial infarction, CABG = coronary artery bypass surgery, CT = computed tomography, PCI = percutaneous coronary intervention, CACS = coronary artery calcium score.

From May 2020 to June 2023, there were 882 patients with AMI. We excluded 334 patients according to the exclusion criteria. Consequently, we included 548 patients as the final study population. The final study population was divided into the high CACS (*n* = 137) and the low–intermediate CACS groups (*n* = 411). The study flow chart is shown in Figure 1.

The comparison of patient clinical characteristics between the high CACS and low–intermediate CACS groups is shown in Table 1. Age was significantly higher in the high CACS group than in the low–intermediate CACS group. The prevalence of shock at admission was significantly higher in the high CACS group than in the low–intermediate CACS group. Hemoglobin levels were significantly lower in the high CACS group than in the low–intermediate CACS group. Estimated GFR was significantly lower in the high CACS group than in the low–intermediate CACS group. Peak creatine kinase (CK) and CK-myocardial band (MB) levels were significantly lower in the high CACS group than in the low–intermediate CACS group. Table 2 shows the comparison of angiographic and procedural findings between the two groups. The number of narrowed coronary arteries was significantly higher, and the prevalence of stenosis at the left main trunk and CTO in non-culprit arteries was significantly higher in the high CACS group than in the low–intermediate CACS group.

**Figure 2:**
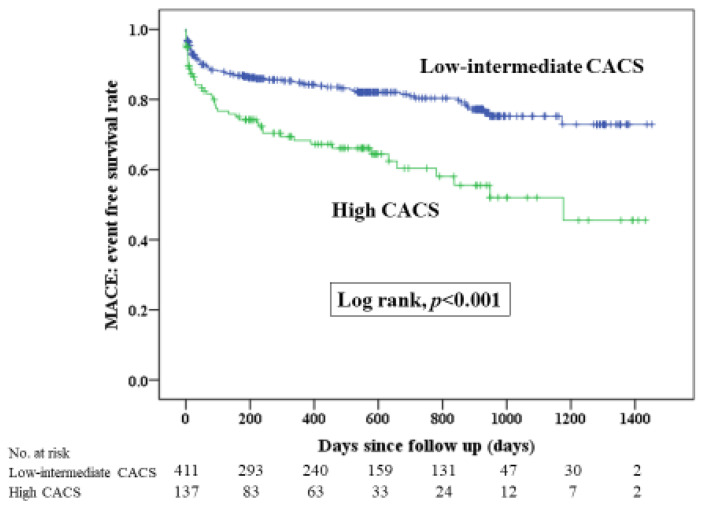
Kaplan–Meier curves for major adverse cardiovascular event-free survival between high CACS and the low–intermediate CACS groups. A log–rank test was used. Abbreviations: MACE = major adverse cardiovascular events, CACS = coronary artery calcium score.

**Figure 3:**
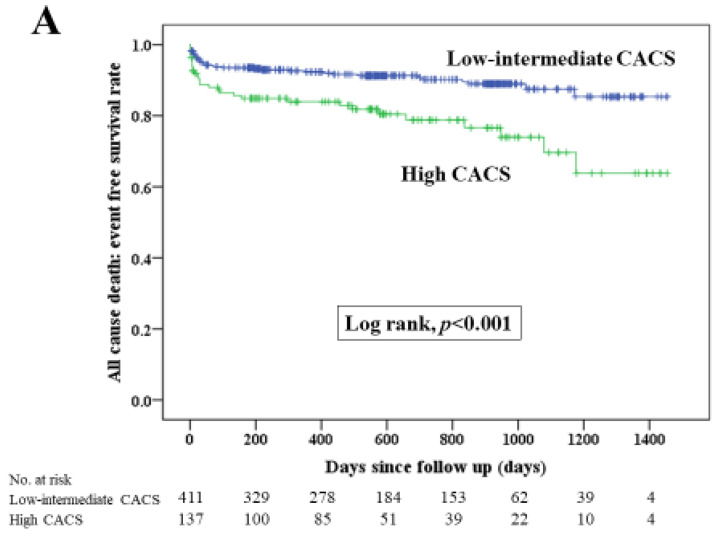

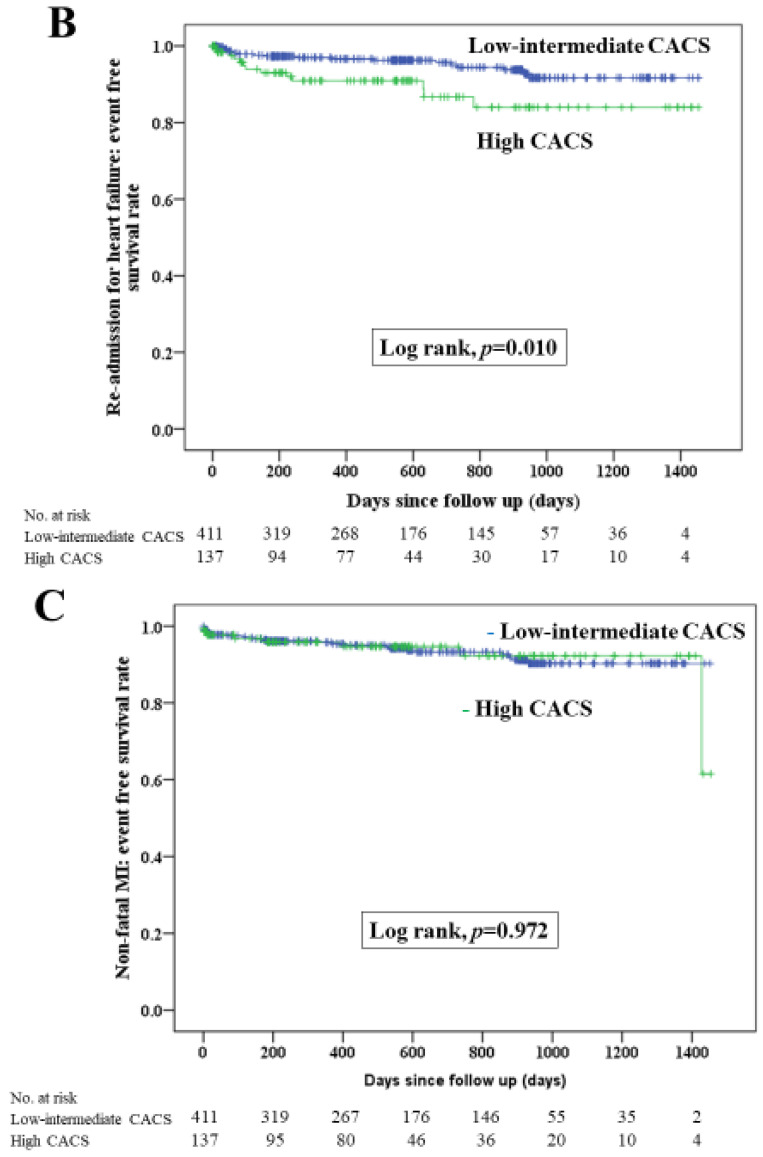

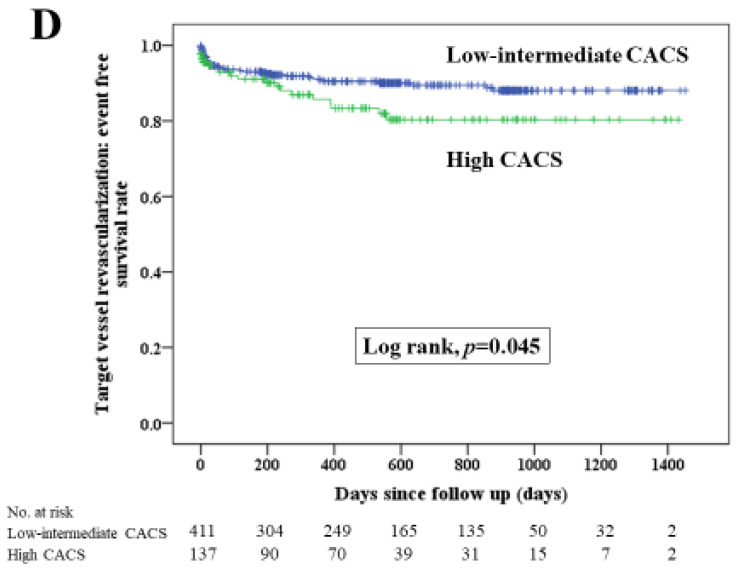
(**A**) Kaplan–Meier curves for all-cause death-free survival between the high CACS and low–intermediate CACS groups. (**B**) Kaplan–Meier curves for re-admission for heart failure-free survival between the high CACS and low–intermediate CACS groups. (**C**) Kaplan–Meier curves for non-fatal MI-free survival between the high CACS and low–intermediate CACS groups (**D**) Kaplan–Meier curves for target vessel revascularization-free survival between the high CACS and low–intermediate CACS groups. A log–rank test was used. Abbreviations: CACS = coronary artery calcium score.

Figure 2 illustrates Kaplan–Meier curves for MACE between the two groups, and Figure 3 shows Kaplan–Meier curves for each component of MACE. The median follow-up duration was 535 days (Q1: 165 days–Q3: 897 days). The incidence of MACE, all-cause death, re-admission for heart failure and target vessel revascularization was significantly greater in the high CACS group than in the low–intermediate CACS group. Table 3 demonstrates the comparison of clinical outcomes between the two groups. The multivariate Cox hazard analysis was performed in Table 4. A high CACS was significantly associated with MACE (HR 1.597; 95% CI 1.081–2.358; *p* = 0.019) after controlling for multiple confounding factors, including age, body weight, chronic renal failure on hemodialysis, shock at admission, hemoglobin levels, platelets, eGFR, 50% ≥ stenosis at left main, first TIMI flow grade, CTO in non-culprit arteries, final PCI procedure and guide-catheter size. All VIF in this multivariate Cox Hazard model were less than 2.5. There was no significant multicollinearity in this analysis.

## 4. Discussion

The main results of the present study are summarized as follows. A total of 548 patients with AMI who underwent PCI were divided into high CACS (*n* = 137) and low–intermediate CACS groups (*n* = 411) according to the CACS derived from non-ECG gated CT. With a median duration of 535 days, MACE were more frequently observed in the high CACS group than in the low–intermediate group. Furthermore, all-cause death, re-admission for heart failure and TVR were more frequently observed in the high CACS group than in the low–intermediate group, whereas the recurrence of non-fatal myocardial infarction was similar between the two groups. The multivariate Cox hazard analysis revealed that a high CACS was significantly associated with MACE (HR 1.597; 95% CI 1.081–2.358; *p* = 0.019) after controlling for multiple confounding factors. Our results suggest the potential of the CACS as a prognostic marker in patients with AMI.

First, we should clarify the differences between the present study and earlier studies. Wayhs et al. reported that asymptomatic patients with a high CACS (CACS > 1000) had more cardiac events than controls [20]. The Multi-Ethnic Study of Atherosclerosis (MESA), a prospective population-based cohort study of 6814 participants without known cardiovascular disease, showed that the CACS was significantly useful for the stratification of risk for future cardiovascular events [21]. A sub-analysis from MESA also revealed that the CACS offered significant improvements in the risk prediction of coronary artery disease [8,22,23]. However, the CACS in these studies was measured by ECG-gated CT when participants were asymptomatic. These studies illustrated the CACS as a tool for primary prevention of cardiovascular events, whereas the subjects in our study were patients with AMI and CACS was measured using non-ECG-gated CT. Some studies confirmed that visual assessment of coronary calcification on non-ECG-gated CT is closely associated with CACS on ECG-gated CT [24,25]. Cheng et al. found that the cumulative calcium score derived from coronary angiography, chest X-rays and echocardiography was associated with long-term outcomes in patients with ST-segment elevation myocardial infarction [26]. Furthermore, Groen et al. calculated the CACS from non-ECG-gated CT and showed that it was correlated strongly with one from ECG-gated CT [27]. However, many previous studies focused on patients with stable angina and did not investigate the association between clinical outcomes and CACS from non-ECG gated CT, whereas our study focused on patients with AMI and investigated the association between clinical outcomes and CACS. Also, the score systems used in previous studies have often been calculated by visual assessment, which required some knowledge and techniques, whereas the CACS used in this study was measured automatically by the application and did not need expertise for calculation.

We should discuss why the high CACS group had worse clinical outcomes than the low–intermediate CACS group. Earlier studies reported that severe calcification of the coronary artery was associated with poor prognosis when patients developed AMI [28], angina [27,29], or even when subjects were asymptomatic [8]. Ishibashi et al. showed that moderate–severe coronary artery calcification in the culprit lesion of AMI detected by coronary angiography was associated with long-term worse clinical outcomes [28]. Kawashima et al. revealed the angiographic coronary artery calcification of patients with angina or with silent ischemia was related to 10-year all-cause mortality [29]. One possible explanation was that heavy coronary artery calcification might imply systemic atherosclerotic disease, which would cause vascular events such as stroke [30]. Another possibility was that patients with severe coronary artery calcification could also have the risk factors for other systemic diseases such as lung cancer [31,32] and chronic kidney disease [33], which would lead to poor clinical outcomes. Our results also showed that the high CACS group had more impaired renal function. Among the four MACE components, only the recurrence of non-fatal myocardial infarction was similar between the high and low–intermediate CACS groups. Although it is difficult to explain the lack of difference in non-fatal myocardial infarction between the two groups, we speculated that coronary artery calcification might be less influential than other factors, such as lipid control, for the prevention of recurrence of AMI. Interestingly, the peak CK and CKP levels were lower in the high CACS group than in the low-intermediate CACS group. Although the mechanism of this result remains unclear, one possibility is that the heavier calcification in coronary arteries represents ischemia for longer periods, which could develop collateral arteries. Those collateral arteries could lessen the infarcted size. In fact, the prevalence of multivessel disease and chronic total occlusion in non-culprit arteries was significantly higher in the high CACS group.

The clinical implications of our study should be noted. Since a high CACS in patients with AMI was associated with worse clinical outcomes, AMI patients with a high CACS should be carefully followed up to prevent future cardiovascular events. Minor symptoms may be signs of major cardiovascular events in these high-risk patients. CACS by non-ECG-gated CT might not be as accurate as one by ECG-gated CT. However, non-ECG-gated CT could be calculated more easily and faster than ECG-gated CT. The CACS by non-ECG-gated CT could be measured even in emergency situations. Other non-invasive modalities, such as cardiac magnetic resonance imaging (MRI), are also useful for predicting clinical outcomes in patients with AMI [34]. The advantages of plain CT are that it is less invasive and easily accessible for scanning, and most hospitals or even some clinics in Japan have CT. Therefore, the CACS by non-ECG-gated CT is easily accessible and may be a simple marker to identify a high-risk group. Since non-ECG-gated CT is accessible more easily in Japan than in other countries, the CACS by non-ECG-gated CT would be useful for predicting clinical outcomes in AMI patients. We do not intend to recommend plain chest CT for patients with AMI, but if patients with AMI underwent plain chest CT for some reason, we can calculate the CACS and might use the CACS as one of the potential prognostic markers.

There are several limitations in the present study. Because this study is a single-center retrospective observational study, there is a potential selection bias. Although the value of each variable’s VIF was less than 2.5, the selection of variables for the multivariate Cox hazard model might be arbitrary in this retrospective study. Although non-ECG-gated chest CT and the CACS calculation are easily accessible, there are fewer necessities to undergo a CT scan for patients with AMI after the pandemic of COVID-19. However, a routine chest CT is useful for patients with AMI because pericardial effusion or vascular complications, such as aortic aneurysm or aortic dissection, is easily understandable. Among other studies regarding the CACS, there are various cutoffs for a high CACS, such as 100, 300, 400 or 1000 [20,21,35], which are quite different from the present study. In the present study, the high CACS group was defined as the highest quantile of all subjects because there are no established cut-off points for a high CACS in non-ECG-gated CT. Although we used 5346 as the cut-off point, we do not intend to suggest 5346 as the definite cut-off point for the high-risk group. Since the image quality of a coronary artery is better in ECG-gated CT than in non-ECG-gated CT, and the CACS in many other studies was calculated by CT with a thickness of 3.0 mm, the absolute value of the CACS would be different between ECG-gated and non-ECG-gated CTs.

## 5. Conclusions

Clinical outcomes were worse in AMI patients with a high CACS than those with a low–intermediate CACS in this retrospective study. A high CACS was significantly associated with MACE after controlling multiple confounding factors in patients with AMI. A high CACS by plain chest non-ECG-gated CT in patients with AMI may be a clinical marker for poor prognosis. However, further studies are warranted to evaluate its validity.

## Figures and Tables

**Table 1:** The comparison of patient clinical characteristics between the high CACS and low–intermediate CACS groups.

	All (*n* = 548)	High CACS Group (*n* = 137)	Low–Intermediate CACS Group (*n* = 411)	*p*-Value
Age, years	72.0 (61.0–79.0)	77.0 (69.5–81.0)	70.0 (59.0–78.0)	<0.001
Male, *n* (%)	424 (77.4)	105 (76.6)	319 (75.2)	0.814
Body weight, kg	63.0 (55.0–71.9)	60.0 (53.0–68.1)	64.0 (55.0–73.0)	0.003
Body mass index (kg/m^2^)	23.7 (21.3–26.0) (*n* = 547)	23.2 (20.8–24.7) (*n* = 136)	23.9 (21.4–26.4)	0.005
Comorbidities				
Hypertension, *n* (%)	418 (76.3)	109 (79.6)	309 (75.2)	0.297
Hyperlipidemia, *n* (%)	268 (49.1) (*n* = 546)	65 (47.4)	203 (49.6) (*n* = 409)	0.657
Diabetes mellitus, *n* (%)	222 (40.7) (*n* = 546)	66 (48.2)	156 (38.1) (*n* = 409)	0.039
Current smoker, *n* (%)	190 (34.8) (*n* = 546)	35 (25.5)	155 (37.9) (*n* = 409)	0.009
Chronic renal failure on hemodialysis, *n* (%)	39 (7.1)	30 (21.9)	9 (2.2)	<0.001
Cardiopulmonary arrest out of hospital, *n* (%)	35 (6.4)	9 (6.6)	26 (6.3)	0.920
Shock at admission, *n* (%)	50 (9.1)	21 (15.3)	29 (7.1)	0.004
Killip class				0.127
Killip class 1 or 2, *n* (%)	412 (75.2)	95 (69.3)	317 (77.1)	
Killip class 3, *n* (%)	64 (11.7)	22 (16.1)	42 (10.2)	
Killip class 4, *n* (%)	72 (13.1)	20 (14.6)	52 (12.7)	
Region of infarction				0.109
Anterior, *n* (%)	288 (52.6)	66 (48.2)	222 (54.0)	
Inferior, *n* (%)	184 (33.6)	54 (39.4)	130 (31.6)	
Posterior, *n* (%)	75 (13.7)	16 (11.7)	59 (14.4)	
Not determined, *n* (%)	1 (0.2)	1 (0.7)	0 (0.0)	
Vital signs at admission				
Systolic blood pressure, mmHg	140 (119–162)	137 (112–158)	141 (120–163)	0.120
Diastolic blood pressure, mmHg	86 (72–100)	78 (66–96)	88 (74–102)	<0.001
Pulse rate, bpm	82 (70–98)	82 (67–100)	82 (70–98)	0.620
Laboratory data				
Hemoglobin levels, g/dL	13.7 (12.1–15.0)	12.5 (11.2–14.1)	14.1 (12.7–15.2)	<0.001
Platelets, ×10^4^/μL	22.2 (18.4–26.3)	19.8 (16.3–24.3)	23.1 (19.2–27.2)	<0.001
Serum creatinine, mg/dL	0.89 (0.71–1.17)	1.03 (0.75–2.31)	0.87 (0.70–1.07)	<0.001
eGFR, mL/min/1.73 m^2^	62.7 (45.7–79.5)	51.0 (22.9–73.5)	64.7 (49.7–80.7)	<0.001
Hemoglobin A1c, %	6.1 (5.7–6.8) (*n* = 542)	6.1 (5.8–6.9) (*n* = 136)	6.0 (5.7–6.8) (*n* = 406)	0.234
C-reactive protein, mg/dL	0.22 (0.09–0.96) (*n* = 545)	0.24 (0.11–1.49) (*n* = 135)	0.21 (0.09–0.92) (*n* = 410)	0.167
Brain natriuretic peptide, pg/mL	138.5 (40.8–537.8) (*n* = 544)	369.5 (120.3–973.7) (*n* = 136)	97.1 (27.9–367.3) (*n* = 408)	<0.001
Peak creatine kinase, U/L	890.5 (237.8–2535.5)	521.0 (164.0–1751.0)	1100.0 (267.0–2783.0)	<0.001
Peak creatine kinase-myocardial band, U/L	76.0 (14.0–253.3)	35.0 (11.0–153.0)	90.0 (17.0–275.0)	0.001
Left ventricular ejection fraction, %	51.0 (39.5–61.1) (*n* = 529)	47.4 (36.6–57.5) (*n* = 131)	53.0 (40.5–62.2) (*n* = 398)	0.001
Medication at admission				
Aspirin, *n* (%)	68 (12.6) (*n* = 539)	32 (23.5) (*n* = 136)	36 (8.9) (*n* = 403)	<0.001
Thienopyridine, *n* (%)	33 (6.1) (*n* = 539)	18 (13.2) (*n* = 136)	15 (3.7) (*n* = 403)	<0.001
Statin, *n* (%)	130 (24.1) (*n* = 539)	50 (36.8) (*n* = 136)	80 (19.9) (*n* = 403)	<0.001
ACE inhibitors or ARBs, *n* (%)	200 (37.2) (*n* = 537)	63 (46.3) (*n* = 136)	137 (34.2) (*n* = 401)	0.011
Beta-blocker, *n* (%)	81 (15.1) (*n* = 537)	44 (32.4) (*n* = 136)	37 (9.2) (*n* = 401)	<0.001
Calcium channel blocker, *n* (%)	199 (37.1) (*n* = 537)	60 (44.1) (*n* = 136)	139 (34.7) (*n* = 401)	0.049
Diuretics, *n* (%)	110 (20.5) (*n* = 537)	51 (37.5) (*n* = 136)	59 (14.7) (*n* = 401)	<0.001
Oral antidiabetic, *n* (%)	143 (26.5) (*n* = 539)	47 (34.6) (*n* = 136)	96 (23.8) (*n* = 403)	0.014
Insulin, *n* (%)	28 (5.2) (*n* = 539)	11 (8.1) (*n* = 136)	17 (4.2) (*n* = 403)	0.079
Direct oral anticoagulants., *n* (%)	21 (3.9) (*n* = 539)	9 (6.6) (*n* = 136)	12 (3.0) (*n* = 403)	0.058
Warfarin, *n* (%)	4 (0.7) (*n* = 539)	3 (2.2) (*n* = 136)	1 (0.2) (*n* = 403)	0.021

Data are expressed as median (Q1–Q3) or numbers (percentages). A Mann–Whitney U test was used for abnormally distributed continuous variables. A Chi-square test was used for categorical variables. Abbreviations: CACS = coronary artery calcium score, PCI = percutaneous coronary intervention, CABG = coronary artery-bypass grafting, eGFR = estimated glomerular filtration rate, ACE inhibitors = angiotensin-converting enzyme inhibitor, ARBs = angiotensin receptor blockers, CKD = chronic kidney disease.

**Table 2:** The comparison of lesion and procedural characteristics between the high CACS and low–intermediate CACS groups.

	All (*n* = 548)	High CACS Group (*n* = 137)	Low–Intermediate CACS Group (*n* = 411)	*p*-Value
Number of narrowed coronary arteries				<0.001
Single, *n* (%)	285 (52.0)	49 (35.8)	236 (57.4)	
Double, *n* (%)	169 (30.8)	50 (36.5)	119 (29.0)	
Triple, *n* (%)	94 (17.2)	38 (27.7)	56 (13.6)	
Infarct-related artery				0.149
Left main-left anterior descending artery, *n* (%)	290 (52.9)	66 (48.2)	224 (54.5)	
Right coronary artery, *n* (%)	183 (33.4)	55 (40.1)	128 (31.1)	
Left circumflex artery, *n* (%)	75 (13.7)	16 (11.7)	59 (14.4)	
Bypass graft, *n* (%)	0 (0.0)	0 (0.0)	0 (0.0)	
Not determined, *n* (%)	0 (0.0)	0 (0.0)	0 (0.0)	
50% ≥ stenosis at left main, *n* (%)	52 (9.5)	23 (16.8)	29 (7.1)	0.001
First TIMI flow grade				0.002
0, *n* (%)	211 (38.5)	34 (24.8)	177 (43.1)	
1, *n* (%)	37 (6.8)	12 (8.8)	25 (6.1)	
2, *n* (%)	90 (16.4)	25 (18.2)	65 (15.8)	
3, *n* (%)	210 (38.3)	66 (48.2)	144 (35.0)	
Final TIMI flow grade				0.824
0, *n* (%)	2 (0.4)	1 (0.7)	1 (0.2)	
1, *n* (%)	6 (1.1)	1 (0.7)	5 (1.2)	
2, *n* (%)	23 (4.2)	6 (4.4)	17 (4.1)	
3, *n* (%)	517 (94.3)	129 (94.2)	388 (94.4)	
CTO in non-culprit arteries, *n* (%)	66 (12.0)	25 (18.2)	41 (10.0)	0.010
Use of aspiration catheter, *n* (%)	29 (5.3)	5 (3.6)	24 (5.8)	0.321
Final PCI Procedure				0.003
POBA only, *n* (%)	19 (3.5)	6 (4.4)	13 (3.2)	
Aspiration only, *n* (%)	2 (0.4)	0 (0.0)	2 (0.5)	
Drug-coated balloon, *n* (%)	21 (3.8)	13 (9.5)	8 (1.9)	
Bare metal stent, *n* (%)	0 (0.0)	0 (0.0)	0 (0.0)	
Drug-eluting stent, *n* (%)	497 (90.7)	116 (84.7)	381 (92.7)	
POBA and aspiration, *n* (%)	7 (1.3)	1 (0.7)	6 (1.5)	
Other, *n* (%)	2 (0.4)	1 (0.7)	1 (0.2)	
Approach site				<0.001
Radial artery, *n* (%)	431 (78.6)	84 (61.3)	347 (84.4)	
Brachial artery, *n* (%)	3 (0.5)	2 (1.5)	1 (0.2)	
Femoral artery, *n* (%)	114 (20.8)	51 (37.2)	63 (15.3)	
Guide-Catheter size (Fr)				<0.001
6Fr, *n* (%)	423 (77.2)	77 (56.2)	346 (84.2)	
7Fr, *n* (%)	121 (22.1)	58 (42.3)	63 (15.3)	
8Fr, *n* (%)	4 (0.7)	2 (1.5)	2 (0.5)	

Data are expressed as numbers (percentages). A Chi-square test was used for categorical variables. Abbreviations: CACS = coronary artery calcium score, TIMI = thrombolysis in myocardial infarction, CTO = chronic total occlusion, PCI = percutaneous coronary intervention, POBA = Plain old balloon angioplasty.

**Table 3:** The comparison of clinical outcomes between the high CACS and low–intermediate CACS groups.

	All (*n* = 548)	High CACS Group (*n* = 137)	Low–Intermediate CACS Group (*n* = 411)	*p*-Value
MACE, *n* (%)	150 (27.4)	58 (42.3)	92 (16.8)	<0.001
All-cause death, *n* (%)	68 (12.4)	29 (21.2)	39 (9.5)	<0.001
Re-admission for heart failure, *n* (%)	32 (5.8)	13 (9.5)	19 (4.6)	0.035
Non-fatal MI, *n* (%)	34 (6.2)	8 (5.8)	26 (6.3)	0.838
Target vessel revascularization, *n* (%)	59 (10.8)	20 (14.6)	39 (9.5)	0.095

Data are expressed as numbers (percentages). A Chi-square test was used for categorical variables. MACE indicate major adverse cardiovascular events: composite of all-cause death, re-admission for heart failure, non-fatal MI and target vessel revascularization. Abbreviations: MACE = major adverse cardiovascular events, CACS = coronary artery calcium score, MI = myocardial infarction.

**Table 4:** Multivariate Cox hazard model to predict MACE.

Composite Endpoint	Hazard Ratio	95% Confidence Interval	*p*-Value
MACE			
Low–intermediate CACS group	Reference		
Unadjusted high CACS group	2.236	1.608–3.108	<0.001
Adjusted high CACS group	1.597	1.081–2.358	0.019
**Component Endpoint**	**Hazard Ratio**	**95% Confidence Interval**	***p*-Value**
All-cause death			
Low–intermediate CACS group	Reference		
Unadjusted high CACS group	2.420	1.496–3.914	<0.001
Adjusted high CACS group	1.173	0.658–2.091	0.589
Re-admission for heart failure			
Low–intermediate CACS group	Reference		
Unadjusted high CACS group	2.451	1.209–4.969	0.013
Adjusted high CACS group	1.576	0.671–3.704	0.297
Non-fatal MI			
Low–intermediate CACS group	Reference		
Unadjusted high CACS group	0.986	0.442–2.198	0.972
Adjusted high CACS group	0.905	0.354–2.315	0.835
Target vessel revascularization			
Low–intermediate CACS group	Reference		
Unadjusted high CACS group	1.723	1.005–2.956	0.048
Adjusted high CACS group	1.595	0.826–3.080	0.164

In the adjusted model, the high CACS group (vs. low–intermediate CACS group) was adjusted for age, body weight, chronic renal failure on hemodialysis, shock at admission, hemoglobin levels, platelets, eGFR, 50% ≥ stenosis at left main, first TIMI flow grade, CTO in non-culprit arteries, final PCI Procedure, guide-catheter size. Abbreviations: MACE = major cardiovascular events, CACS = coronary artery calcium score, eGFR = estimated glomerular filtration rate, TIMI = thrombolysis in myocardial infarction, CTO = chronic total occlusion, PCI = percutaneous coronary intervention.

## Data Availability

The data that support the findings of this study are available from the corresponding author upon reasonable request.
